# Satiating Effect of High Protein Diets on Resistance-Trained Individuals in Energy Deficit

**DOI:** 10.3390/nu11010056

**Published:** 2018-12-28

**Authors:** Justin Roberts, Anastasia Zinchenko, Krishnaa T. Mahbubani, James Johnstone, Lee Smith, Viviane Merzbach, Miguel Blacutt, Oscar Banderas, Luis Villasenor, Fredrik T. Vårvik, Menno Henselmans

**Affiliations:** 1 School of Psychology and Sport Science, Cambridge Centre for Sport and Exercise Sciences, Anglia Ruskin University, East Road, Cambridge CB1 1PT, UK; james.johnstone@anglia.ac.uk (J.J.); lee.smith@anglia.ac.uk (L.S.); viviane.merzbach@anglia.ac.uk (V.M.); 2 Department of Biochemistry, Kings College, University of Cambridge, Kings Parade, Cambridge CB2 1ST, UK; a.zinchenko@live.de; 3 International Scientific Research Foundation for Fitness and Nutrition, 1073 LC Amsterdam, The Netherlands; miguelblacutt@hotmail.com (M.B.); oscarbanderastk@gmail.com (O.B.); luis@villasenor.net (L.V.); ftvaarvik@gmail.com (F.T.V.); menno.henselmans@gmail.com (M.H.); 4 Department of Surgery, Addenbrookes Hospital, Cambridge CB2 0QQ, UK; ktam2@cam.ac.uk

**Keywords:** dietary protein, satiety, ghrelin, peptide YY, resistance training

## Abstract

Short-term energy deficit strategies are practiced by weight class and physique athletes, often involving high protein intakes to maximize satiety and maintain lean mass despite a paucity of research. This study compared the satiating effect of two protein diets on resistance-trained individuals during short-term energy deficit. Following ethical approval, 16 participants (age: 28 ± 2 years; height: 1.72 ± 0.03 m; body-mass: 88.83 ± 5.54 kg; body-fat: 21.85 ± 1.82%) were randomly assigned to 7-days moderate (PRO_MOD_: 1.8 g·kg^−1^·d^−1^) or high protein (PRO_HIGH_: 2.9 g·kg^−1^·d^−1^) matched calorie-deficit diets in a cross-over design. Daily satiety responses were recorded throughout interventions. Pre-post diet, plasma ghrelin and peptide tyrosine tyrosine (PYY), and satiety ratings were assessed in response to a protein-rich meal. Only perceived satisfaction was significantly greater following PRO_HIGH_ (67.29 ± 4.28 v 58.96 ± 4.51 mm, *p* = 0.04). Perceived cravings increased following PRO_MOD_ only (46.25 ± 4.96 to 57.60 ± 4.41 mm, *p* = 0.01). Absolute ghrelin concentration significantly reduced post-meal following PRO_MOD_ (972.8 ± 130.4 to 613.6 ± 114.3 pg·mL^−1^; *p* = 0.003), remaining lower than PRO_HIGH_ at 2 h (−0.40 ± 0.06 v −0.26 ± 0.06 pg·mL^−1^ normalized relative change; *p* = 0.015). Absolute PYY concentration increased to a similar extent post-meal (PRO_MOD_: 84.9 ± 8.9 to 147.1 ± 11.9 pg·mL^−1^, PRO_HIGH_: 100.6 ± 9.5 to 143.3 ± 12.0 pg·mL^−1^; *p* < 0.001), but expressed as relative change difference was significantly greater for PRO_MOD_ at 2 h (+0.39 ± 0.20 pg·mL^−1^ v −0.28 ± 0.12 pg·mL^−1^; *p* = 0.001). Perceived hunger, fullness and satisfaction post-meal were comparable between diets (*p* > 0.05). However, desire to eat remained significantly blunted for PRO_MOD_ (*p* = 0.048). PRO_HIGH_ does not confer additional satiating benefits in resistance-trained individuals during short-term energy deficit. Ghrelin and PYY responses to a test-meal support the contention that satiety was maintained following PRO_MOD_, although athletes experiencing negative symptoms (i.e., cravings) may benefit from protein-rich meals as opposed to over-consumption of protein.

## 1. Introduction

Weight loss is an essential component of success for many strength athletes, as well as individuals involved in general strength training. Weight class athletes restrict dietary energy intake typically for 7–21 days prior to a competition [1], or during phases of training, to enhance the strength to body-mass ratio, improve body composition, and increase the competitive advantage in weight class events [2]. For physique/ bodybuilding athletes, similar practices of caloric restriction often occurs for longer time periods i.e., >12 weeks to reach a very low body fat percentage, often going below 5% to improve aesthetical appearance and succeed in bodybuilding competitions [2,3]. During dieting phases many athletes may substantially increase their protein intake, as this practice has been shown to be beneficial to maintain lean mass whilst reducing body-fat [4,5,6,7,8,9,10,11,12,13]. Although the recommended daily intake of protein for healthy adults has been indicated at 0.83 g·kg^−1^·d^−1^ ([14] with habitual protein intakes being reportedly greater at ~1.2 g·kg^−1^·d^−1^ [15]), protein requirements for resistance-trained individuals to achieve maximal fractional synthetic rate and strength performance may exceed these recommendations ranging from 1.3 to 1.8 g·kg^−1^·d^−1^ [16,17]. During acute energy deficit, protein requirements may be subtly higher at 1.8–2.0 g·kg^−1^·d^−1^ to offset lean muscle losses. There is, however, controversy as to whether substantially higher protein intakes ranging from 2.3 to 3.1 g·kg^−1^·d^−1^ may in fact yield optimal results [13] with evidence that resistance-trained athletes consume protein intakes as high as 4.3 g·kg^−1^·d^−1^ [18].

Higher protein intakes may also provide an additional benefit of increased satiety. A recent meta-analysis by Dhillon et al. (2016) concluded that higher protein meals (ranging from 97 to 188 g) increased fullness ratings more than lower protein meals [19]. However, this analysis focused on short-term studies (up to 10 h) in untrained individuals and can therefore not necessarily be extrapolated to longer-term satiety over several days/ weeks. There may be a possibility that a person becomes accustomed to a high protein intake and that a further increase in protein intake does not result in additional satiety. This point can be particularly important for strength athletes who generally consume a higher protein diet beyond the recommended levels for maximal performance [16,17].

Very few long-term, randomized, controlled trials have examined satiety and *ad libitum* energy intake in normal weighted individuals following a low-protein vs a high-protein diet [20,21,22]. Only three studies have been conducted on resistance-trained individuals showing that a higher protein intake may be beneficial for muscle retention and well-being following a weight loss diet [4,6,23]. Walberg et al. have shown that even though a higher protein diet (1.6 g·kg^−1^·d^−1^) during an extreme weight loss intervention (18 kcal·kg^−1^) led to positive nitrogen balance compared to a lower protein group (0.8 g·kg^−1^·d^−1^); it also led to reduced maximal isometric muscular endurance in weight lifters [6]. In a different study, 2.3 g·kg^−1^·d^−1^ protein intake was shown to be beneficial for muscle retention in 40% energy deficit compared to the 1.0 g·kg^−1^·d^−1^ control group [4]. However, higher protein intake was also associated with increased fatigue for participants in this study, although this has been contested elsewhere [23]. 

The finding of increased fatigue is particularly important considering that many athletes increase their protein intake during caloric restriction in order to limit muscle loss, reduce hunger and increase the feeling of satiety [13]. An increase in fatigue can result in decreased motivation to train [4], reduced training efficiency and thus have a negative effect on athletic performance [6] particularly during the competition preparation phase, in which many weight-class strength athletes undergo weight loss interventions. It is important to note that the control condition examined in this study [4] represented an unusually low protein intake (1.0 g·kg^−1^·d^−1^) for strength athletes that is below the established recommendations [16]. Equally so, very high protein intakes, exceeding these recommendations, may not only negatively impact on an athletes’ well-being and reduce the effort given to training, but also carries the risk of displacement of other nutrients. 

Therefore, the aim of this study was to compare the satiating effect of two diets with a different protein content in resistance-trained subjects in energy deficit, participant well-being and training motivation. A moderately high protein diet (PRO_MOD_) with an intake corresponding to the recommendation for maximal performance (1.8 g·kg^−1^·d^−1^) [16,17] and a very high protein diet (PRO_HIGH_) with a protein intake of 2.9 g·kg^−1^·d^−1^ were used as main interventions. It was hypothesised that a protein intake of 2.9 g·kg^−1^·d^−1^ would not impact on satiety levels more so than an intake of 1.8 g·kg^−1^·d^−1^ but may reduce the motivation to train.

## 2. Materials and Methods 

### 2.1. Participants

This study was conducted in accordance with the Declaration of Helsinki, and the protocol was approved by the Faculty of Science and Technology Ethics Committee, Anglia Ruskin University (approval number: FST/FREP/15/595). A priori sample size based on G*power software (α = 0.05 and 1-β = 0.80) using satiety data from Mettler et al. (2010) [4] based on resistance training individuals consuming 2.3 g·kg^−1^·d^−1^ vs. 1 g·kg^−1^·d^−1^, estimated 14 in each group. Participants were required to have a resistance training background of >6 months, while actively training >3 h per week, in a similar manner to previous research [4]. This was to ensure participants were undergoing habitual training at the point of study inclusion, and beyond the time-frame typically employed during acute training studies [24,25,26,27,28,29].

Informed consent was obtained from all individual participants prior to study inclusion. All participants satisfactorily completed a health screen questionnaire, and had no known history of cardiovascular or metabolic abnormalities (e.g., diabetes); or recent viral infections or injuries which would prevent them from maintaining habitual training sessions. As part of the inclusion criteria, participants were required to not have any blood related disorders, no known eating disorders and no known adverse reactions to whey protein supplementation, gluten or coconut oil. Additionally, all participants were required to not be taking any medication/supplementation which could influence satiety levels. Lacto-vegetarians were eligible for the study.

Twenty-two individuals (13 men, 9 women) volunteered for study inclusion. However, five participants withdrew prior to first dietary intervention (due to personal factors conflicting with the study requirements) and one participant did not complete the final assessment, resulting in 16 resistance trained participants in this randomised, controlled trial (9 men, 7 women). None of the participants reported using any anabolic drugs, and were required to refrain from taking additional supplementation (e.g., creatine, beta-alanine) for 4 weeks prior to and during the study. Participant characteristics are shown in Table 1.

### 2.2. Pre-Intervention Measures

All testing took place within the Cambridge Centre for Sport and Exercise Science, Human Physiology Laboratory, Anglia Ruskin University, Cambridge. Prior to main trial procedures, participants were required to visit the laboratory ~72–96 h prior to start of the first and second trials to ascertain body composition measures required for energy intake calculations. As such, participants were requested to arrive acutely fasted (i.e., no food within 3 h of assessment, and maintain habitual hydration patterns) with last consumption of fluid (~0.5 L water) 1 h prior to assessment to standardise procedures. Body-mass (Seca 799, Hamburg, Germany), height (Seca CE123 stadiometer, Hamburg, Germany) and body composition were assessed under temperature controlled conditions.

Following a standardized 5-min resting period in a supine position, single frequency bioelectrical impedance (Impedimed DF50, Carlsbad, CA, USA) was undertaken for initial assessment of body composition measures, with particular emphasis on hydration indices (to confirm adherence to the pre-testing requirements) and phase angle (a proxy marker of muscle quality to confirm trained status (with >7.0 reported for female athletes, and >8.0 for male athletes from previous research [30]). In addition, estimated body fat was evaluated using an 8 site skinfold calliper assessment (using guidelines outlined by the International Society for the Advancement of Kinanthropometry (ISAK)). All body composition measures were undertaken by the same researcher. Within the same timeframe, participants completed habitual food intake and training diaries.

### 2.3. Dietary Assessment

As part of pre-intervention measures, and throughout the intervention, participants were requested to maintain habitual food/activity diaries (following individual guidance in diary collation, with emphasis on meal content, portion size and weight, and fluid intake) using the smart phone app/browser program (www.MyFitnessPal.com). This method has been shown to be a reliable food tracking method used in previous studies [31,32,33]. Diaries were assessed using Nutritics Professional Dietary Analysis software (Nutritics Ltd., Co. Dublin, Ireland). Initial assessment was used to monitor typical food choices and habitual caloric balance (including criterion assessment of training levels). From this, assessment of individual maintenance caloric intake was undertaken using the formula of Katch-McArdle (1996) based on an estimated resting daily energy expenditure (RDEE) of 370 + (21.6 * lean body weight in kg) and adjusted against training requirements and non-exercise adaptive thermogenesis [34], based on previous research [35].

### 2.4. Experimental Design and Intervention Measures

This study employed an experimental, randomised controlled, counter-balanced, crossover design. Participants were randomly assigned to a 7-day matched calorie-restricted diet (20% from estimated maintenance calories) of either moderate (1.8 g·kg^−1^·d^−1^) or high (2.9 g·kg^−1^·d^−1^) total protein intake (PRO_MOD_ and PRO_HIGH_ respectively) with a fixed frequency of 4 meals per day. Guidance on meal intake, food options in line with habitual patterns and portion size was provided to each participant, along with provision of additional whey protein to supplement daily intake where required. Participants visited the laboratory prior to starting the dietary intervention (day 0), and again at on the completion of the 7 days (day 8) for assessment of satiety measures to a test meal (see below). After each diet, participants were required to undertake a 3-day period in which the same meal frequency (4 meals per day) was maintained, and protein intake was fixed (1.8 g·kg^−1^·d^−1^). However, energy, carbohydrate and fat intake was permitted *ad libitum* at each meal until participants felt full. Perception of satiety was noted by participants across each day during this period. This was included to determine the dieting condition less likely to result in acute post-diet overeating. 

Throughout this 10-day period, participants recorded all food and fluid intakes using an individually allocated MyFitnessPal account, and maintained habitual training patterns. Compliance of dietary intake was checked daily by the research team, and further individual support was provided as required to meet expected intakes. At the end of the first dietary period, participants undertook a washout period and returned to habitual intake patterns for 4 weeks before returning to carry out the opposing dietary condition. Mean dietary intakes at baseline and for each intervention are shown in Table 2.

### 2.5. Laboratory Measures

Laboratory assessment took place on day 0 and 8 of each main intervention period, in addition to pre-trial body composition measures. Participants were instructed to refrain from strenuous physical activity 24–48 h prior to all laboratory visits, arrive strictly overnight fasted (>12 h) and avoid any undue exertion travelling to the laboratory. Upon arrival, participants were required to rest in a seated position for ~30 min without any undue distractions and complete an online satiety questionnaire (see below). Following this, a venous whole blood sample (T0) was collected from participants by a qualified phlebotomist into duplicate 4 mL K3EDTA vacutainers (Greiner Bio-One GmbH, Kremsmunster, Austria). Once collected, stabilisers were added to each of the samples prior to being centrifuged for 15 min at 3000 g. The plasma layer was pipetted and aliquoted into sterile, non-pyrogenic, polypropylene cryovials (Fisherbrand, Fisher Scientific, Loughborough, UK) and immediately frozen at −20 °C for later assessment of satiety hormones: ghrelin and peptide YY (PYY). Body composition measures (height, weight, bioelectrical impedance) were assessed at the end of each 7-day intervention period as previously described.

### 2.6. Test Meal and Satiety Measures

At each visit for both intervention conditions, following resting measures, participants consumed a standardised high-protein breakfast meal (Asda Scottish porridge oats (Asda Stores Ltd., Leeds, UK) comprising (per 100g): 367 kcal; 12.0 g protein; 61.0 g carbohydrate; 6.2 g fat), added whey protein (Pure Protein GF-1, USN UK Ltd., Longbridge, Birmingham, UK), coconut oil (Asda 100% raw extra virgin coconut oil, Asda Stores Ltd., Leeds, UK) with 200 mL water under supervision based on individual estimated caloric requirements for the PRO_HIGH_ condition and a protein content of 0.725 g·kg^−1^). On immediate completion of the test meal, a stopwatch was started and participants captured their subjective post-prandial satiety responses using a previously validated satiety/hunger questionnaire based on a standard 100 mm visual analogue scale [36,37] at 15, 30, 60, 90 and 120 min. The questionnaire was based on immediate response to set questions (“How ‘hungry’ do you feel?”; “How ‘full’ do you feel?”; “How ‘satisfied’ do you feel?”; “How ‘much’ can you eat right now?”). Participants were required to remain in a quiet, seated position during this time with no activity except light reading, and moving to a designated couch (supine position) for collection of blood samples at T60 and T120 (minutes) for assessment of selected satiety hormones. Data was normalised to individual relative change and relative change differences in a similar manner to blood analyses (see Section 2.8 below).

### 2.7. Post-Laboratory Measures and Support

An online satiety questionnaire was additionally completed at the end of each day across the 7-day intervention, and during the post-diet 3-day *ad libitum* period. The questionnaire captured individual perceived responses to the following questions: (i) how hungry were you today?, (ii) how full were you today?, (iii) how satisfied were you today with your diet?, (iv) how much do you think you can eat right now?, (v) how high have your food cravings been today?, (vi) how often did you feel energetic and active today?, (vii) how often were you in a good mood today?, (viii) how much did you enjoy your training today?, (ix) how much do you feel you can give your best effort at training today?. Mean data was collated for days 1–3 and 5–7 across the intervention period, and for days 1–3 of the *ad libitum* period. To increase dietary compliance during each intervention period, participants were provided with additional whey protein (Pure Protein GF-1, whey protein concentrate/ soya protein isolate, USN UK Ltd., Longbridge, Birmingham, UK) comprising (per 100g): 370 kcal; 71 g protein; 9.8 g carbohydrate; 4.4 g fat.

### 2.8. Biochemical Analyses

All samples were analysed at the Department of Surgery, Addenbrookes Hospital, Cambridge. Plasma samples with 4-(2-Aminoethyl) benzenesulfonyl fluoride hydrochloride (AEBSF; Sigma, Dorset, UK) to a final concentration of 1 mg·mL^−1^ were frozen to −20 °C within 30 min of centrifuging (15 min at 3000 g). Samples were subsequently thawed aseptically in a 37 °C water bath, centrifuged at 3000 g for 5 min to sediment any cryoprecipitates and then analysed in duplicate using a human total ghrelin ELISA kit or human PYY ELISA kit (EMD Millipore Corporation, Billerica, MA, USA) for ghrelin and PYY measurements according to manufacturers’ instructions. ELISA plates were read using a FLUOstar OPTIMA plate reader (BMG Labtech, Aylesbury, UK). In addition to raw concentrations (pg·mL^−1^), data were individually normalised as relative change (to reflect normalised post-intervention effects) and relative change differences (to reflect normalised intervention effects taking into consideration pre-intervention results) according to the following equations:

Relative change (relative Δ pg·mL^−1^) = $\frac{x-y}{x}$ where *x* = pre-meal resting sample, *y* = post-meal respective sample time-points (i.e., 60, 120 min);

Relative change difference (relative Δ difference pg·mL^−1^) = $\left(\frac{x_{post}-y_{post}}{x_{post}}\right)-\left(\frac{x_{pre}-y_{pre}}{x_{pre}}\right)$ where pre = pre-intervention results, post = post-intervention results.

### 2.9. Statistical Analyses

Statistical analyses were performed using SPSS (v24, IBM, Armonk, NY, USA). Dependent variable distributions were assessed for normality using a Shapiro-Wilk test as well as manual inspections of M-estimators, histograms, stem-and-leaf plots and boxplots. Potential order and treatment effects were assessed prior to main analyses using a paired samples t-test. Baseline participant characteristics (gender) were assessed using an independent samples t-test. A mixed design repeated measures ANOVA (diet, time) was performed for main analyses, with Bonferonni post-hoc comparisons where applicable. Where pertinent, relative change questionnaire scores were assessed using a paired samples t-test. An alpha level of ≤0.05 was employed for statistical significance. Data are reported as mean ± S.E.

## 3. Results

### 3.1. Nutrition Intake and Body Composition Data

Mean dietary intake across both interventions is shown in Table 2. Prior to starting the first intervention average caloric intake for all participants was significantly greater (2379.81 ± 201.48 kcal·d^−1^, F = 5.07, *p* = 0.013, ηp^2^ = 0.25) in comparison to main intervention intakes, although similar to predicted habitual intakes when taking into consideration rest days (2381.25 ± 131.31 kcal·d^−1^). Across intervention periods, targeted caloric deficit was achieved in relation to estimated maintenance calories (based on individual requirements for both training and rest days) for both PRO_MOD_ (−22.9 ± 1.0%) and PRO_HIGH_ (−21.0 ± 1.1%) conditions.

Whilst protein was generally maintained during PRO_MOD_ compared with pre-intervention intakes, PRO_HIGH_ resulted in an expected increase in relative protein from 1.84 ± 0.15 to 2.89 ± 0.01g·kg^−1^·d^−1^ (F = 78.29_(diet x time)_, *p* < 0.0001, ηp^2^ = 0.84). Post-intervention protein intakes were significantly different between conditions (*p* < 0.0001), with mean intakes demonstrating excellent compliance with target amounts. Relative carbohydrate intake was reduced during PRO_HIGH_ by 25.37 ± 6.16% from 2.55 ± 0.16 g·kg^−1^·d^−1^ to 1.90 ± 0.16g·kg^−1^·d^−1^ (F = 11.54_(diet x time)_, *p* = 0.004, ηp^2^ = 0.44), and was significantly lower than PRO_MOD-post_ (*p* < 0.0001). To meet caloric deficit requirements, during both interventions total fat intake was significantly reduced by 39.42 ± 5.76% (*p* < 0.0001) and 27.63 ± 6.35% (*p* < 0.0001) for PRO_MOD_ and PRO_HIGH_ respectively (F = 4.64_(diet x time)_, *p* = 0.048, ηp^2^ = 0.24). Following interventions, no differences for fat intake were reported between conditions (*p* > 0.05). However, relative fat intakes pre-intervention were significantly higher for PRO_MOD_ compared to PRO_HIGH_ (*p* = 0.008).

Mean body-mass significantly decreased within both interventions (F = 24.00_(time)_, *p* < 0.0001, ηp^2^ = 0.62) by 1.28 ± 0.30% with PRO_MOD_ (from 88.37 ± 5.42 to 87.18 ± 5.31 kg, *p* = 0.001) and by 1.49 ± 0.36% with PRO_HIGH_ (from 88.48 ± 5.54 to 87.14 ± 5.42 kg, *p* = 0.002) demonstrating compliance with acute energy deficit based on estimated maintenance calories. This corresponded with a significant within-condition reduction in phase angle (F = 26.02_(time)_, *p* < 0.0001, ηp^2^ = 0.63) for both PRO_MOD_ (−0.41 ± 0.09°, *p* < 0.0001) and PRO_HIGH_ (−0.29 ± 0.12°, *p* = 0.04). Short-term energy deficit resulted in a reduction in hydration indices, with both total body water (PRO_MOD_: 53.14 ± 1.81 to 51.16 ± 1.59%, *p* = 0.002; PRO_HIGH_: 53.04 ± 1.70 to 51.24 ± 1.80%, *p* = 0.018) and intracellular water (PRO_MOD_: 59.07 ± 0.75 to 57.88 ± 0.67%, *p* = 0.001; PRO_HIGH_: 58.89 ± 0.71 to 57.88 ± 0.81%, *p* = 0.035) decreasing within-condition. It was noted that mean training frequency was comparable between dietary conditions (PRO_MOD_: 6.38 ± 0.63 sessions; PRO_HIGH_: 7.00 ± 0.50 sessions, *p* = 0.44) across the 10-day intervention period.

### 3.2. Dietary Intervention Perceived Satiety Responses

Across both dietary interventions, participants maintained a daily satiety questionnaire as previously described. Data, based on absolute values (using a 100 mm visual analogue scale), were averaged for the beginning (days 1–3) and end of each intervention (days 5–7). Mean responses are shown in Table 3. Perception of satisfaction was significantly greater at the end of PRO_HIGH_ (F = 4.52_(diet)_, *p* = 0.05, ηp^2^ = 0.23) compared with PRO_MOD_ over days 5–7 (67.29 ± 4.28 mm v 58.96 ± 4.51 mm respectively, *p* = 0.04). Participants also reported a significant mean increase in perception of cravings (F = 5.93_(time)_, *p* = 0.028, ηp^2^ = 0.28) within PRO_MOD_ only from days 1–3 (46.25 ± 4.96 mm) to days 5–7 (57.60 ± 4.41 mm, *p* = 0.01). No other differences were reported between conditions, including perceived training enjoyment and motivation to train. 

### 3.3. Test-Meal Satiety Hormone Responses

*Absolute values:* Mean plasma ghrelin and PYY concentrations in response to the test-meal are shown in Table 4. No differences were reported for pre- or post-intervention concentrations between conditions (*p* > 0.05) for either analyte prior to consuming the test-meal. However, both ghrelin and PYY were notably lower (albeit non-significant, *p* = 0.06 and *p* = 0.11 respectively) at T0 following PRO_MOD_ compared with pre-intervention data. Plasma ghrelin reduced as expected by T60 in all assessments, but typically increased by T120 (F = 28.77_(time)_, *p* < 0.001, ηp^2^ = 0.63), remaining significantly lower than pre-meal concentrations (*p* ≤ 0.036). However, following PRO_MOD_, plasma ghrelin continued to decrease to 613.57 ± 114.26pg·mL^−1^ by T120 (*p* = 0.003 within condition compared to T0) and was significantly different overall compared to pre intervention responses (*p* = 0.015). Across all assessments, plasma PYY significantly increased from T0 to T120 (F = 33.05_(time)_, *p* < 0.001, ηp^2^ = 0.70), with no differences reported between pre- or post-intervention concentrations.

**Figure 1:**
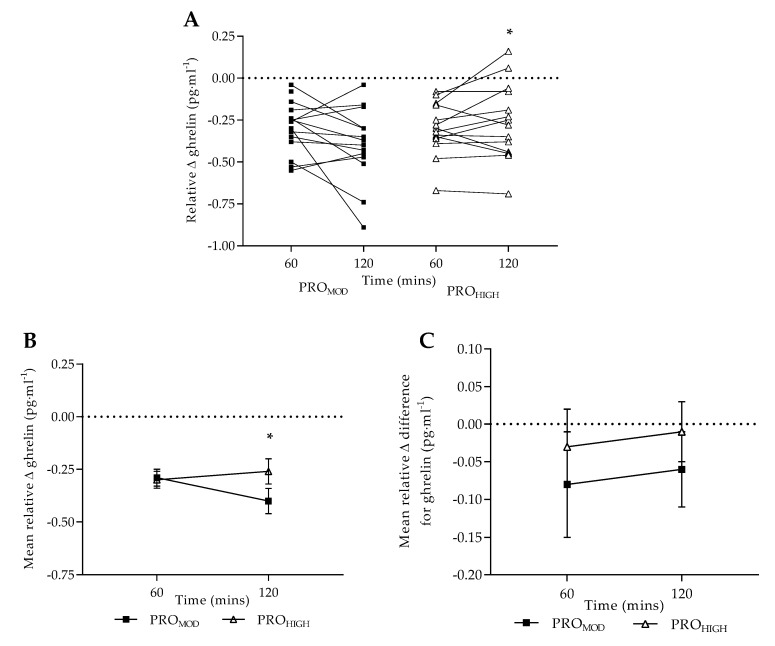
Plasma ghrelin concentrations following test-meal (pg·mL^−1^; mean ± SE): (**A**) individual values expressed as normalised relative change to baseline levels; (**B**) mean values expressed as normalised relative change to baseline levels; (**C**) mean relative difference change (taking into consideration pre-intervention results). PRO_MOD_ PRO_HIGH_ denote moderate and high protein conditions. Dashed line provides reference point to pre-meal normalisation. * denotes significant difference overall to PRO_MOD_ at corresponding time-point (*p* = 0.015).

**Figure 2:**
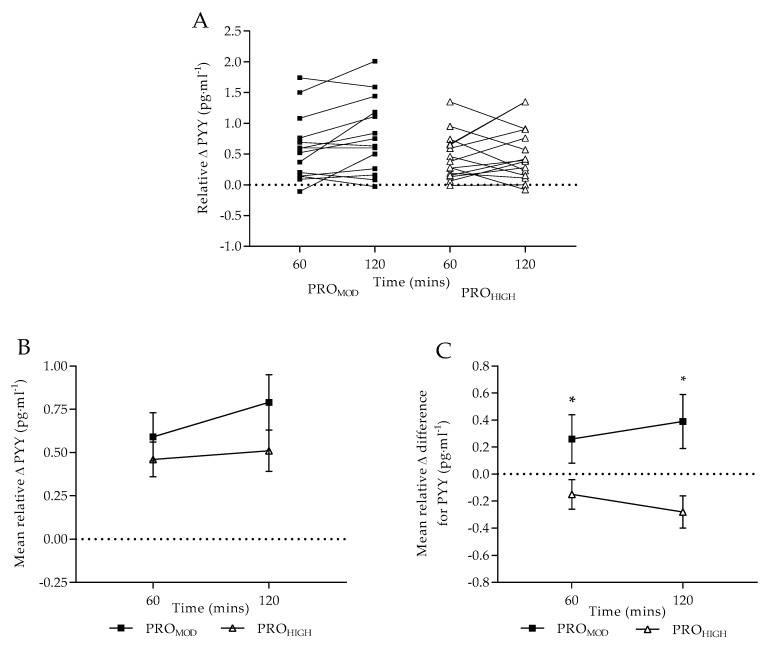
Plasma peptide YY concentrations following test-meal (pg·mL^−1^; mean ± SE): (**A**) individual values expressed as normalised relative change to baseline levels; (**B**) mean values expressed as normalised relative change to baseline levels; (**C**) mean relative difference change (taking into consideration pre-intervention results). PRO_MOD_ and PRO_HIGH_ denote moderate and high protein conditions. Dashed line provides reference point to pre-meal normalisation. * denotes significant difference between dietary conditions at each time-point (*p* ≤ 0.018).

Relative values: Plasma ghrelin and PYY expressed as normalized relative change (individual results and mean data, reflective of intervention diet effect) and normalized relative change difference (mean data, reflective of overall change in comparison to habitual intake) are shown in Figure 1 and Figure 2 respectively. Normalized relative change in ghrelin concentration was significantly lower (F = 5.90_(diet × time)_, *p* = 0.029, ηp^2^ = 0.30) at T120 following PRO_MOD_ (−0.40 ± 0.06 pg·mL^−1^) compared to PRO_HIGH_ (−0.26 ± 0.06 pg·mL^−1^, *p* = 0.015), with no prior differences reported at T60 between interventions (*p* > 0.05). Relative change difference in ghrelin concentration was comparable between conditions (*p* > 0.05). 

Normalized relative change for PYY increased from 0.59 ± 0.14 pg·mL^−1^ at T60 to 0.79 ± 0.16 pg·mL^−1^ at T120 with PRO_MOD_, but was not deemed significant (*p*=0.07, main effect for time) in comparison to PRO_HIGH_ (0.46 ± 0.10 pg·mL^−1^ at T60 to 0.51 ± 0.12 pg·mL^−1^ at T120). However, relative change difference for PYY was significantly greater for PRO_MOD_ at both T60 (0.26 ± 0.18 pg·mL^−1^) and T120 (0.39 ± 0.20 pg·mL^−1^) compared with negative findings for PRO_HIGH_ at the same time-points (T60: −0.15 ± 0.11 pg·mL^−1^, T120: −0.28 ± 0.12 pg·mL^−1^; *p* ≤ 0.018).

### 3.4. Test-Meal Satiety Questionnaire Responses

**Figure 3:**
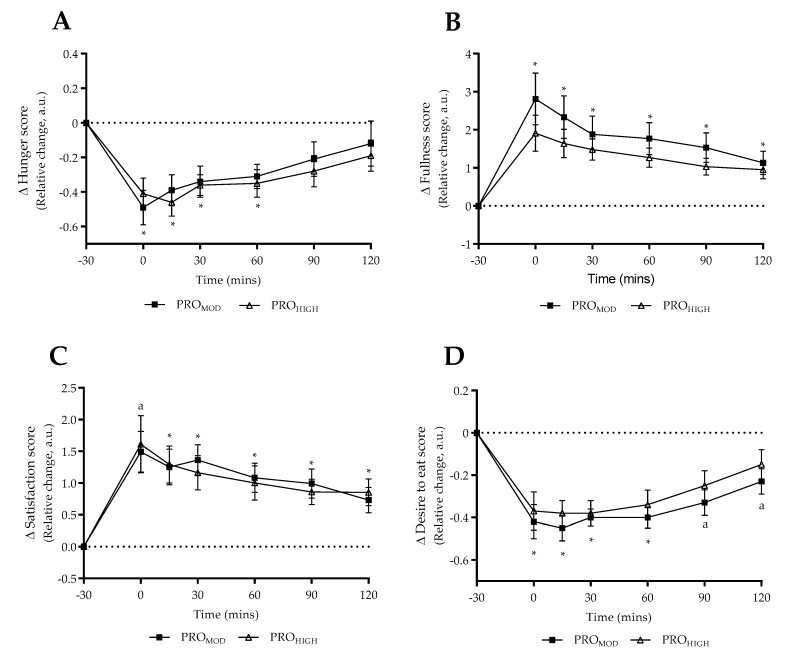
Questionnaire responses in relation to test meal under laboratory conditions. Data expressed as relative change (post diet, normalised) for both moderate (PRO_MOD_) and high (PRO_HIGH_) protein conditions (mean ± SE). (**A**) Perception of hunger; (**B**) Perception of fullness; (**C**) Perception of satisfaction; (**D**) Perception of desire to eat. Responses in relation to pre-meal (−30 min) time-point. 0 min denotes immediate completion of test meal. Dashed line provides reference point to pre-meal perceived state. ^*^ denotes significant difference compared to pre-meal time-point (*p* ≤ 0.05) within both dietary conditions. ^a^ denotes significant difference compared to pre-meal time-point for PRO_MOD_ only (*p* ≤ 0.048). No differences reported between dietary conditions.

Satiety questionnaire responses to the test-meal following each intervention (normalized relative change) are shown in Figure 3. Perception of hunger was significantly reduced (F = 15.34_(time)_, *p* < 0.0001, ηp^2^= 0.51) for 60 min post test-meal following both interventions (*p* ≤ 0.024) with no differences reported between diets (*p* > 0.05). In a similar pattern, perception of fullness significantly increased post-meal (F = 16.52_(time)_, *p* < 0.0001, ηp^2^ = 0.52) and remained above pre-meal values at T120 for both interventions (*p* ≤ 0.05), with no differences reported between diets (*p* > 0.05). Interestingly, following immediate completion of the test-meal satisfaction was only significantly increased with PRO_MOD_ (*p* = 0.006), beyond which perception of satisfaction remained elevated across all time-points within each dietary condition (F = 13.65_(time)_, *p* < 0.0001, ηp^2^ = 0.48). Perception of desire to eat reduced following consumption of the test meal as expected (F = 16.90_(time)_, *p* < 0.0001, ηp^2^ = 0.53) and remained lower than pre-meal values for 60 min in both interventions (*p* ≤ 0.02). However, perception of desire to eat only remained blunted at T90 and T120 within-PRO_MOD_ only (*p* ≤ 0.048). 

**Figure 4:**
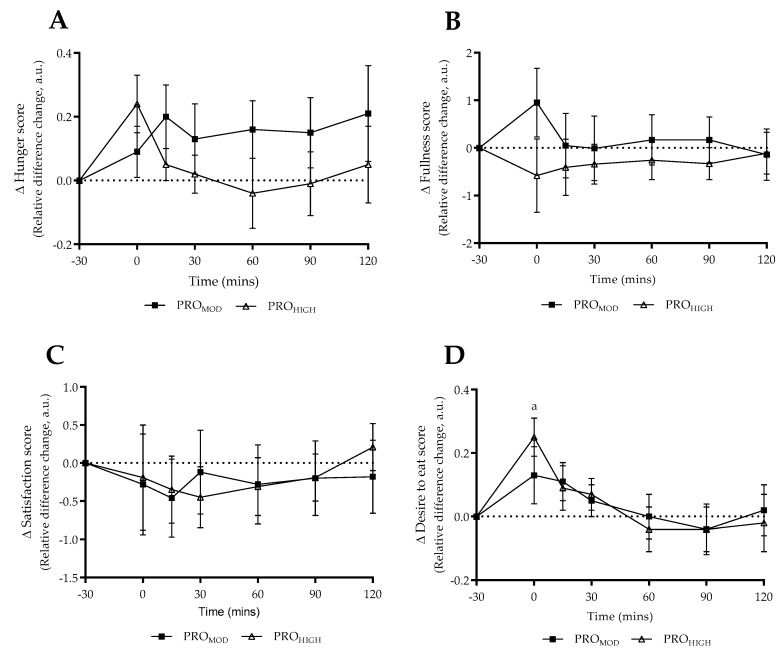
Questionnaire responses in relation to test meal under laboratory conditions. Data expressed as relative difference change (baseline to post diet) for both moderate (PRO_MOD_) and high (PRO_HIGH_) protein conditions (mean ± SE). (**A**) Perception of hunger; (**B**) Perception of fullness; (**C**) Perception of satisfaction; (**D**) Perception of desire to eat. Responses in relation to pre-meal (−30 min) time-point. 0 min denotes immediate completion of test meal. Dashed line provides reference point to pre-meal perceived state. ^a^ denotes difference to pre-meal time-point within PRO_HIGH_ only (*p* = 0.028). No differences between dietary conditions observed.

Satiety questionnaire responses to the test-meal expressed as normalized relative change difference (taking into consideration pre-intervention responses) are shown in Figure 4. No significant differences were report within or between dietary conditions for perception of hunger, fullness or satisfaction (*p* > 0.05). For perception of desire to eat, a significant main effect for time was reported (F = 5.43_(time)_, *p* < 0.0001, ηp^2^ = 0.27), with post-hoc analysis demonstrating a significant increase on immediate completion of the test-meal for PRO_HIGH_ only (*p* = 0.028).

### 3.5. Ad libitum Intake and Satiety Responses

Mean dietary intake during the *ad libitum* phase following each dietary intervention is shown in Table 5. Despite higher absolute energy and fat intakes with PRO_HIGH_, no significant differences were reported between conditions for any variable (*p* > 0.05). Compliance with target protein intake was met for both conditions. Satiety responses across days 1-3 of the *ad libitum* phase are shown in Table 6, along with delta scores in comparison to days 5-7 of the previous intervention. Perception of hunger was significantly reduced following transition to the *ad libitum* period (F = 5.18_(time)_, *p* = 0.038, ηp^2^ = 0.26) within PRO_MOD_ only (−9.67 ± 4.68 mm, *p* = 0.046). Likewise, perception of fullness (F = 10.33_(time)_, *p* = 0.006, ηp^2^ = 0.41) and satisfaction (F = 5.72_(time)_, *p* = 0.03, ηp^2^ = 0.28) were significantly improved within-condition relative to the end of the PRO_MOD_ diet (fullness: 9.78 ± 3.74 mm, *p* = 0.015; satisfaction: 13.11 ± 4.33 mm, *p* = 0.005). Perceived desire to eat also reduced on transition to an *ad libitum* phase (F = 14.86_(time)_, *p* = 0.002, ηp^2^ = 0.50) for PRO_MOD_ only (−16.78 ± 4.54 mm, *p* = 0.001). 

A significant interaction effect was reported for perceived cravings (F = 5.00_(dietxtime)_, *p* = 0.041, ηp^2^ = 0.25), with post-hoc analysis indicating a significant reduction for PRO_MOD_ (−13.00 ± 4.27 mm, *p* = 0.004). The relative change in perceived cravings for PRO_MOD_ was significantly reduced in comparison to PRO_HIGH_ (*p* = 0.04) following transition to an *ad libitum* phase. Participants reported improvements in general mood within-condition (*p* = 0.05) only. Perceived motivation to train was reportedly improved with PRO_MOD_ (F = 4.52_(time)_, *p* = 0.05, ηp^2^ = 0.23) by 8.97 ± 5.08mm (*p* = 0.05). No other differences were reported between dietary conditions during the *ad libitum* phase (*p* > 0.05).

## 4. Discussion

The main finding from this study was that the perceived satiating effect of two diets with different protein content was generally comparable in resistance-trained participants undergoing a period of acute energy deficit. Therefore, consuming more protein during acute energy deficit does not appear to improve perceived satiety, as reported elsewhere [38,39,40,41,42]. However, when considering hormonal adaptations to an acute dietary intervention in response to a protein-rich test-meal, a moderate protein, energy deficit diet (1.8 g·kg^−1^·d^−1^) was deemed more satiating when compared to a high protein (2.9 g·kg^−1^·d^−1^) diet. These results suggest that whilst in acute energy deficit, a PRO_MOD_ diet may be sufficient to maintain training requirements in accordance with previous recommendations [5,43,44]. It is, however, important to note that a PRO_HIGH_ diet did not appear to disadvantage participants and may offer individual benefits discussed below.

Across the 7-day intervention period, participant responses to the daily satiety questionnaire were largely similar for all categories, with the exception of both cravings and satisfaction (Table 3). By the end of the dietary intervention, whilst consuming a PRO_MOD_ diet, participants reported increased cravings, which was not experienced to the same extent following PRO_HIGH_. Additionally, perceived satisfaction was not only maintained during PRO_HIGH_, but was significantly greater than PRO_MOD_ at the end of the intervention period. Similar findings in response to a high protein meal have been observed elsewhere [45]. This implies that during short-term caloric deficit a substantial increase in protein ratio beyond recommended levels for maximal lean muscle gain may be better sustained by resistance trained athletes who are more prone to cravings. Participant well-being, mood, training enjoyment and motivation to train were comparable between dietary conditions. As training frequency was equally maintained across dietary conditions, this inferred that an increase in protein intake beyond 1.8 g·kg^−1^·d^−1^ did not compromise training responses, and did not result in reduced motivation or perceived energy during training as hypothesized.

In the current study subjects maintained their habitual training routine without any external motivation that was intended to increase their performance. As exercise fatigue has been reported to increase during acute caloric deficit [4], it is possible that participants in the current study subconsciously decreased training effort, despite maintaining session frequency. Interestingly, phase angle (PA) assessed via bioelectrical impedance indicated a significant reduction following both dietary conditions, more notably with PRO_MOD_. PA has been reported to be a proxy measure of muscle “quality” and is associated with sex, age, body mass index (BMI) and fat mass percentage (FM%) [30,46,47,48]. A decrease in PA during both dietary conditions may indicate unfavorable changes to muscle integrity as a result of energy restriction. However, the comparable reduction in hydration indices likely infers decreased PA was transient based on acute energy deficit. Our previous findings demonstrated an increase in PA when resistance trainees underwent a period of supervised intensive training to volitional exhaustion during short-term energy deficit when consuming a PRO_HIGH_ diet [30]. It is feasible, therefore, that in order to maintain muscle integrity during periods of acute caloric deficit more intensive training intensity and substantially increased protein intake is required, which is supported elsewhere [13].

Plasma ghrelin responses were comparable with previous research [45,49,50,51,52]. However, in response to a laboratory test meal, notable differences were reported between dietary conditions. Following PRO_MOD_, plasma ghrelin remained significantly reduced at 2 h post-meal indicating a sustained satiating effect. When data was considered as normalized relative change (Figure 1B), this pattern was highlighted further with differences reported between dietary conditions in response to a protein-rich test meal. Furthermore, as no differences were observed between dietary conditions when taking into consideration pre-intervention ghrelin responses (Figure 1C), this would suggest that the satiating effect following PRO_MOD_ was associated with the higher protein content of the test meal.

It should be noted that baseline protein intake for all participants was nearly identical to the protein requirements for the PRO_MOD_ condition. Therefore, the sustained reduction in 2-h ghrelin response observed following PRO_MOD_ may also be related to the energy balance of the individual (both a reduction in caloric and fat intake) across the intervention period [53,54]. It is also noteworthy, that following PRO_MOD_, plasma ghrelin was reduced at T0 compared with pre-intervention levels which presented an interesting trend (*p* = 0.06, ηp^2^ = 0.22) considering the reported increase in cravings within-condition by day 7. As plasma ghrelin followed expected responses pre and post PRO_HIGH_ [55], the findings indicate that satiety may be acutely enhanced by the consumption of a high-protein meal during periods of energy deficit when following a PRO_MOD_ approach. Individuals experiencing periodic cravings when following a PRO_MOD_ diet may therefore benefit by increasing the protein content of individual meals when required [56] without necessarily over-consuming protein. 

This may be important considering the potential risks of longer-term consumption of very high protein intakes in some individuals (i.e., those with existing, or predisposition for, kidney disease [57]), and current recognition that high protein diets modulate intestinal microbiota production of tryptophan, leading to elevated metabolites (e.g., indoxyl sulphate) acting as uremic toxins [58,59]. There is also current suggestion that high protein diets may negatively impact intestinal function, potentially via bacterial metabolites produced as a result of undigested proteins [60]. High protein diets, in combination with reduced carbohydrate/fiber intake may reduce short-chain fatty acid concentrations, impacting on colonic environment/mucosa [15] which may influence individual health in the longer term. This supports the contention that a PRO_MOD_ approach may be sufficient for resistance-trained individuals during energy deficit.

On completion of the dietary interventions, absolute plasma PYY did not significantly differ between conditions in response to a standardized test meal, as observed elsewhere [49]. The expected increase in plasma PYY [52] in response to a protein-rich test meal was consistent, with a similar rate of change from T0-T60, following both dietary conditions. It is, however, noteworthy that following PRO_MOD_, a greater rate of change in plasma PYY from T60-T120 was evident (*p* = 0.07), which is comparable with the sustained satiating effect observed with plasma ghrelin. When data were compared as normalized relative change (Figure 2B), no differences were reported between dietary conditions (*p* = 0.06) potentially explained by the mixed individual responses observed. However, when taking into consideration pre-intervention PYY responses (Figure 2C), an overall positive response was observed for PRO_MOD_ at both T60 and T120 in contrast to PRO_HIGH_. This infers that a PRO_MOD_ intervention improved acute satiety to a greater extent than PRO_HIGH_. Collectively, these results suggest that individuals may become accustomed to a higher protein intake, and therefore chronic consumption of a PRO_HIGH_ diet may lose its satiating effect over time [61].

Various factors modulate PYY concentrations, including acute/chronic energy balance, as well as nutrient composition of a meal. High protein meal content has been shown to elicit greater PYY secretion leading to short-term sensations of fullness [62,63]. PYY is an agonist at neuropeptide Y2 receptors and increasing levels leads to both reduced hunger perception and food intake [62,64]. In the current study, nutrient composition of the test-meals remained the same across all testing sessions. Therefore, although absolute changes in PYY were not reportedly different between dietary interventions, the relative change difference indicated that both the decrease in caloric intake, along with the higher protein content of the test meal resulted in acute increases in PYY concentrations over the 2-h monitoring period for PRO_MOD_. Although not significant, the observed increase in absolute PYY concentration for PRO_HIGH_ at T0 may be explained by the increase in dietary protein over the 7-days. However, the hormonal response to a protein-rich meal for PRO_HIGH_ was not considered different to pre-intervention patterns. This further supports the contention that a moderate protein diet favorably improved acute physiological satiety in contrast to very high protein intakes.

Perceived satiety responses to the protein-rich test meal were largely comparable between dietary conditions in contrast to physiological responses. Only “desire to eat” remained significantly reduced until 2 h post-meal with PRO_MOD_ (Figure 3D), which in part supported the satiating effect observed for plasma ghrelin. However, this was not the case for perceived hunger, fullness or satisfaction. Whilst perceived satisfaction was comparable between conditions beyond T15, it was noted that participants reported being more satisfied on completion of the test-meal with PRO_MOD_ (T0, Figure 3C). However, as the mean values at T0 were similar between conditions, it was noted that a trend towards significance was observed for PRO_HIGH_ (*p* = 0.06) indicating that perceived responses to the test-meal were likely comparable for satisfaction. Overall, our results support previous findings that circulating levels of ghrelin and PYY do not correlate with the subjective perception of appetite [52,65]. 

Based on the acute energy deficit period undertaken it is feasible that perceived responses may not have changed dramatically compared to habitual intake. Interestingly when test-meal questionnaire responses were normalized in relation to pre-intervention results (Figure 4), no differences were reported between dietary conditions. The only within-condition finding at T0 for “desire to eat” for PRO_HIGH_ (Figure 4D), may be explained by the fact that the protein content of the test-meal was based on the higher protein intervention. Therefore, following a 7-day high protein (2.9 g·kg^−1^·d^−1^) energy deficit period, it is likely that participants had attained a degree of habituation to the protein intake and hence experienced a greater “desire to eat” on completion of the test-meal. This is, in part, supported by the hunger and fullness scores at T0, albeit non-significant.

On completion of the 7-day dietary intervention, participants then completed a 3-day period in which protein intake was fixed at 1.8 g·kg^−1^·d^−1^, with *ad libitum* consumption of carbohydrate, fat and energy intake until perceived fullness. This was included to provide insight as whether the preceding intervention increased the likelihood of over-eating on cessation of short-term energy deficit. Although energy and fat intakes were reportedly greater following PRO_HIGH_, no differences were reported between groups during the *ad libitum* phase, as similarly observed elsewhere [64]. Despite having followed a monitored period of reduced (20%) energy intake, participants tended to only partially increase caloric intake mainly through increased total fat closer to pre-intervention levels. This suggests, at least in the short-term, that resistance-trained athletes are unlikely to over-consume calories on completion of an energy deficit period. This may reflect the tendency to follow habitual dietary intake patterns during periods of training. 

In a similar manner, the satiety questionnaire responses during the *ad libitum* period were comparable between dietary conditions. However, when relative responses were compared to the end of the previous intervention period, the change from a PRO_MOD_ approach resulted in significant within-condition improvements in hunger, fullness, satisfaction, desire to eat, as well as general participants’ mood and motivation to train. Notably, the reduction in cravings observed following PRO_MOD_ was significantly different to PRO_HIGH_. Collectively this suggests that whilst a short-term PRO_MOD_ energy deficit period may result in improved hormonal satiety responses, periodic inclusion of higher protein intakes may support sustained periods in which athletes undergo caloric deficit. Alternatively, athletes undergoing periods of energy deficit may physiologically benefit from a PRO_MOD_ approach, but should be mindful of increasing protein content (either based on single meals, or as a dietary approach) when perceived cravings or a reduction in mood and/or training motivation occurs.

On completion of the study, several limitations were noted. Firstly, that in monitoring habitual dietary intake a longer lead in period would have provided clearer insight. All participants were resistance-trained individuals experienced in maintaining individual macronutrient ratios (i.e., 1.8 g·kg^−1^·d^−1^) and followed regular training routines. Dietary records were kept in the days leading into each testing period prior to each intervention. As such, the observed habitual intake (Table 2) included the requested rest days and therefore did not reflect maintenance intakes expected when taking into consideration training days over a typical week (i.e., 2628 ± 144 kcal·d^−1^). However, based on the period collected, energy intakes were comparable to expected intakes for less active periods (e.g., 2381 ± 131 kcal·d^−1^) for this cohort (comprising both male and female athletes). Therefore, although the habitual intakes were comparable prior to each intervention phase, they likely do not reflect accurate mean energy intakes. However, the decrease in body-mass observed across each intervention, along with monitored reduced energy intakes, indicated that participants achieved the 20% energy deficit as expected.

Although training frequency was maintained across both intervention periods, training intensity was not monitored. Therefore, although perceived energy, training motivation and training enjoyment was not different between dietary conditions, it is difficult to know whether participants maintained overall training intensity. It is feasible, as previously mentioned, that fatigue was offset through a subconscious reduction in training intensity during the energy deficit period. Anecdotally, most participants reported that the PRO_HIGH_ diet was challenging (i.e., reduced mental clarity) towards the end of the intervention phase, possibly indicating that such diets may be poorly tolerated in the longer term. Careful attention to monitoring training intensity, along with perceived well-being responses during periods of acute energy deficit may be warranted to ensure performance maintenance.

Another limitation observed was that other gut hormones (i.e., glucagon-like peptide 1 (GLP-1) and cholecystokinin (CCK)) were not assessed in this study. This would have supported our findings in determining that a PRO_MOD_ diet had a greater acute satiating effect after consumption of a high protein meal. GLP-1 for example is secreted in conjunction with PYY, and has been associated with appetite response and decreased food intake [62,66,67,68]. An increased GLP-1 response to PRO_MOD_ would provide clearer insight that the satiating effect of the test-meal was related to localized stomach/intestinal responses to a protein-rich breakfast meal, and confirmed our reasoning that athletes may become accustomed to chronic consumption of a high protein diet. Likewise, adjunct measurements of CCK released from the duodenum in response to meal consumption influences satiety acutely [62]. Along with reduced ghrelin responses, measures of CCK would have supported the contention that PRO_MOD_ resulted in greater satiety in this cohort.

Whilst the results from this study indicate that a PRO_MOD_ preferentially influences hormonal responses to a test-meal, it is evident that perceived responses were comparable between intervention diets. Further research is therefore warranted to assess whether perceived satiety responses are different when undertaking prolonged energy deficit periods. This is pertinent considering previous research has indicated that fasting ghrelin levels may be increased in response to longer term energy deficit [69,70]. Furthermore, with current recognition that high protein diets (including type of dietary protein) likely modulate gut microbiota, this may have inference to both neurotransmitter and satiety responses (e.g., GLP-1), especially considering inter-individual variance [15]. 

Although the sample size in the current study was deemed sufficient based on a priori power assessment (comparable to previous research [4]), determination of satiety responses in larger resistance-trained cohorts is also warranted. In addition, with the observation that athletes may benefit from consuming a PRO_MOD_ diet during energy deficit, but periodically include protein-high meals to offset negative symptoms (i.e., cravings), assessment of nutritional periodization during periods of caloric deficit would be beneficial to ascertain sustainability to such diets. Finally, maintenance of training performance and lean gains during periods of acute or chronic energy deficit should be considered to determine whether a PRO_MOD_ diet sustains beneficial responses in resistance-trained athletes. 

## 5. Conclusions

During acute energy deficit in resistance-trained individuals, consuming protein intakes in excess of recommended athlete guidelines did not improve overall perceived satiety in comparison to more habitual, moderate protein intakes. Furthermore, a PRO_MOD_ diet favorably improved hormonal responses to a test-meal compared with a PRO_HIGH_ diet. Therefore, as long as training motivation/ performance is not compromised during short-term energy deficit, moderate protein intakes (1.8 g·kg^−1^·d^−1^) are likely to be adequate for resistance-trained individuals. The findings also suggest that where individuals experience negative symptoms (i.e., cravings), implementation of periodic high protein meals may be sufficient to maintain satisfaction, and diet sustainability.

## Figures and Tables

**Table 1:** Participant characteristics and baseline measurements.

Variable	All Participants (n = 16)	Male (n = 9)	Female (n = 7)
Age (years)	28 ± 2	26 ± 2	30 ± 3
Height (m)	1.72 ± 0.03	1.79 ± 0.02	1.63 ± 0.04 *
Body-mass (kg)	88.83 ± 5.54	95.68 ± 5.73	80.01 ± 6.21
Body-fat (%)	21.85 ± 1.82	18.33 ± 1.91	26.37 ± 2.57 *
BIA FM (kg)	25.52 ± 3.37	23.12 ± 3.02	28.60 ± 6.82
BIA FM (%)	27.53 ± 2.46	23.40 ± 1.99	32.84 ± 4.40 *
BIA FFM (kg)	63.31 ± 3.50	72.56 ± 3.05	51.41 ± 3.48 *
BIA FFM (%)	72.47 ± 2.46	76.60 ± 1.99	67.16 ± 4.40 *
PA (°)	8.27 ± 0.16	8.71 ± 0.10	7.70 ± 0.19 *
TBW (%)	52.26 ± 1.77	55.23 ± 1.43	48.43 ± 3.17 *
ICW (%)	58.49 ± 0.81	59.48 ± 0.48	57.23 ± 1.43
ECW (%)	41.48 ± 0.81	40.52 ± 0.84	42.71 ± 1.45

Data presented as mean ± SE. Body-fat (%) refers to estimation via skinfold measures. BIA denotes bioelectrical impedance analysis. FM = fat-mass; FFM = fat-free mass; PA = phase angle; TBW = total body water; ICW = intracellular water; ECW = extracellular water. Hydration markers from BIA. * denotes significantly different to male cohort, *p* ≤ 0.05.

**Table 2:** Mean dietary intake at baseline and across intervention periods.

Variable	Category	PRO_MOD_		PRO_HIGH_	
		PRE	POST	PRE	POST
Energy Intake	(kcal·d^−1^)	2359.44 ± 220.84 *	2057.44 ± 104.47	2270.71 ± 150.41 *	2119.44 ± 122.79
	(kcal·kg^−1^·d^−1^)	26.89 ± 1.94	23.83 ± 0.93	25.53 ± 1.17	24.35 ± 0.84
Protein Intake	(g·d^−1^)	158.19 ± 13.55	162.94 ± 10.14	166.71 ± 18.33	256.13 ± 16.64 ^a,b^
	(g·kg^−1^·d^−1^)	1.80 ± 0.12	1.84 ± 0.02	1.84 ± 0.15	2.89 ± 0.01 ^a,b^
	(%EI)	27.79 ± 1.98	31.68 ± 1.33	28.79 ± 2.13	48.44 ± 1.93 ^a,b^
Carbohydrate Intake	(g·d^−1^)	220.75 ± 25.34	238.75 ± 13.32	221.79 ± 12.07	160.88 ± 13.88 ^a,b^
	(g·kg^−1^·d^−1^)	2.57 ± 0.25	2.81 ± 0.18	2.55 ± 0.16	1.90 ± 0.16 ^a,b^
	(%EI)	38.08 ± 2.52	46.55 ± 1.32 ^a^	40.28 ± 2.45	30.32 ± 1.82 ^a,b^
Fat Intake	(g·d^−1^)	90.94 ± 11.40	46.88 ± 2.62 ^a^	73.79 ± 7.68	47.31 ± 2.80 ^a^
	(g·kg^−1^·d^−1^)	1.02 ± 0.11	0.55 ± 0.03 ^a^	0.83 ± 0.08 ^b^	0.55 ± 0.03 ^a^
	(%EI)	14.83 ± 0.77	9.12 ± 0.25 ^a^	12.86 ± 0.90 ^b^	8.94 ± 0.21 ^a^

Data presented as mean ± SE, and for macronutrient categories expressed in grams per day, grams per kg per day, and percentage of energy intake (EI). PRO_MOD_ denotes moderate protein condition (target: 1.80 g·kg^−1^·d^−1^); PRO_HIGH_ denotes high protein condition (target: 2.90 g·kg^−1^·d^−1^). * denotes main effect for time (*p* = 0.04), but no significant post-hoc findings. ^a^ denotes significantly different to PRE within condition (*p* ≤ 0.005). ^b^ denotes significantly different to PRO_MOD_ at same time-point (*p* ≤ 0.024).

**Table 3:** Satiety and well-being questionnaire responses during dietary intervention phases.

Question	PRO_MOD1–3_	PRO_MOD5–7_	PRO_HIGH1–3_	PRO_HIGH5–7_
Hunger	48.54 ± 3.48	51.46 ± 4.89	43.33 ± 4.22	41.67 ± 3.68
Fullness	62.29 ± 4.11	58.65 ± 4.64	64.38 ± 3.81	65.52 ± 3.99
Satisfaction	65.42 ± 4.38	58.96 ± 4.51	68.65 ± 2.34	67.29 ± 4.28 ^a^
Desire to eat	50.94 ± 5.53	57.92 ± 5.45	45.00 ± 5.13	46.15 ± 5.58
Cravings	46.25 ± 4.96	57.60 ± 4.41 ^b^	40.83 ± 4.59	47.19 ± 4.60
Energy	65.00 ± 2.81	67.81 ± 4.54	64.48 ± 3.18	69.06 ± 4.64
Mood	72.92 ± 3.35	67.92 ± 5.33	67.71 ± 3.26	71.88 ± 3.89
Training enjoyment	72.29 ± 4.35	68.23 ± 4.73	71.67 ± 4.48	73.02 ± 4.19
Training motivation	71.67 ± 4.97	66.56 ± 4.68	70.21 ± 3.93	70.83 ± 5.92

Data represent mean scores over days 1–3 and days 5–7 during moderate (PRO_MOD_) or high (PRO_HIGH_) diet periods. Data based on arbitrary units (a.u.) from participant responses using a visual analogue scale (VAS, 0–100 mm) and presented as mean ± SE. Question categories paraphrased. ^a^ denotes significant difference to PRO_MOD5–7_ (*p* = 0.04). ^b^ denotes significant increase within group compared to days 1–3 (*p* = 0.01).

**Table 4:** Mean plasma ghrelin and peptide YY concentrations in response to test meal.

Hormone	Time (mins)	PRO_MOD_		PRO_HIGH_	
		PRE	POST	PRE	POST
Ghrelin (pg·mL^−1^)	0	1125.97 ± 125.85	972.81 ± 130.42	920.12 ± 143.43	1088.17 ± 158.77
	60	696.42 ± 96.61 *	659.73 ± 86.39 *	672.27 ± 119.29 *	786.61 ± 117.33 *
	120	758.91 ± 129.38 *	613.57 ± 114.26 *^, a^	714.75 ± 136.56 *	850.60 ± 147.68 *
PYY (pg·mL^−1^)	0	103.62 ± 10.15	84.87 ± 8.94	87.94 ± 11.45	100.65 ± 9.54
	60	130.83 ± 9.09 *	129.38 ± 10.49 *	133.84 ± 15.14 *	141.02 ± 12.23 *
	120	137.60 ± 8.71 *	147.14 ± 11.94 *	142.11 ± 13.29 *	143.34 ± 11.98 *

Data presented as mean ± SE. PRO_MOD_ denotes moderate protein condition; PRO_HIGH_ denotes high protein condition. Time-point 0 refers to resting concentrations pre-feeding; other time-points refer to corresponding time post-breakfast meal. * denotes significant differences to 0 time-point within condition only (*p* ≤ 0.036). ^a^ denotes overall difference to pre intervention responses (*p* = 0.015).

**Table 5:** Mean dietary intake during *ad libitum* period.

Variable	Category	PRO_MOD_	PRO_HIGH_
Energy Intake	(kcal·d^−1^)	2197.81 ± 177.58	2348.14 ± 232.46
	(kcal·kg^−1^·d^−1^)	25.64 ± 1.62	26.52 ± 1.81
Protein Intake	(g·d^−1^)	162.38 ± 11.18	165.07 ± 12.89
	(g·kg^−1^·d^−1^)	1.85 ± 0.03	1.86 ± 0.07
	(%EI)	30.87 ± 2.13	29.40 ± 1.89
Carbohydrate Intake	(g·d^−1^)	214.94 ± 19.61	230.61 ± 24.33
	(g·kg^−1^·d^−1^)	2.57 ± 0.24	2.65 ± 0.23
	(%EI)	39.14 ± 1.98	39.66 ± 1.85
Fat Intake	(g·d^−1^)	72.69 ± 9.71	81.29 ± 11.01
	(g·kg^−1^·d^−1^)	0.84 ± 0.09	0.90 ± 0.09
	(%EI)	12.64 ± 0.76	13.29 ± 0.70

Data presented as mean ± SE, and for macronutrient categories expressed in grams per day, grams per kg per day, and percentage of energy intake (EI). PRO_MOD_ denotes post moderate protein condition; PRO_HIGH_ denotes post high protein condition. During *ad libitum* phase, meal frequency set to 4 meals, and target protein intake of 1.80 g·kg^−1^·d^−1^, otherwise unrestricted. No significant differences reported between conditions.

**Table 6:** Questionnaire responses during *ad libitum* dietary phase.

Question Category	PRO_MOD_	PRO_HIGH_	ΔPRO_MOD_	ΔPRO_HIGH_
Hunger	41.67 ± 3.61	40.78 ± 3.48	−9.67 ± 4.68 ^a^	−0.89 ± 2.74
Fullness	69.00 ± 4.39	73.39 ± 3.10	9.78 ± 3.74 ^a^	7.87 ± 4.61
Satisfaction	73.11 ± 3.80	71.56 ± 3.00	13.11 ± 4.33 ^a^	4.26 ± 4.89
Desire to eat	39.67 ± 2.96	38.03 ± 3.62	−16.78 ± 4.54 ^a^	−8.12 ± 5.22
Cravings	42.89 ± 4.12	46.15 ± 4.23	−13.00 ± 4.27 ^a^	−1.04 ± 5.08 *
Energy	71.33 ± 3.31	71.93 ± 2.91	2.99 ± 4.30	2.87 ± 3.34
Mood	76.11 ± 4.03	77.55 ± 2.09	8.33 ± 3.70 ^a^	5.68 ± 2.72 ^a^
Training enjoyment	79.35 ± 4.44	74.67 ± 4.51	9.23 ± 6.57	1.67 ± 4.43
Training motivation	78.85 ± 5.39	72.89 ± 4.99	8.97 ± 5.08 ^a^	1.33 ± 3.93

Data represent mean scores over days 1–3 during *ad libitum* post diet period. Data based on arbitrary units (a.u.) from participant responses using a visual analogue scale (VAS) and presented as mean ± SE. Δ scores are relative to end of intervention diet. Question categories paraphrased. ^a^ denotes significant difference within group compared to end of intervention diet (*p* ≤ 0.05).* denotes significant difference to ΔPRO_MOD_ (*p* = 0.04).
