# Correction: Roberts et al. “Satiating Effect of High Protein Diets on Resistance-Trained Individuals in Energy Deficit” *Nutrients* 2019, 11(1), 56

**DOI:** 10.3390/nu11071543

**Published:** 2019-07-08

**Authors:** Justin Roberts, Anastasia Zinchenko, Krishnaa Mahbubani, James Johnstone, Lee Smith, Viviane Merzbach, Miguel Blacutt, Oscar Banderas, Luis Villasenor, Fredrik T. Vårvik, Menno Henselmans

**Affiliations:** 1 Cambridge Centre for Sport and Exercise Sciences, School of Psychology and Sport Science, Anglia Ruskin University, East Road, Cambridge CB1 1PT, UK; 2 Kings College, Kings Parade, University of Cambridge, Cambridge CB2 1ST, UK; 3 International Scientific Research Foundation for Fitness and Nutrition, 1073 LC Amsterdam, The Netherlands; 4 Department of Surgery, Addenbrookes Hospital, Cambridge CB2 0QQ, UK

The authors wish to make a correction to the published version of their paper [1]. In preparing a later manuscript, it was noted that the equations under Section 2.8 Biochemical Analyses should have reflected the post-meal hormone concentrations in relation to the pre-meal values (as undertaken during the analysis stage). Corrected versions of the relative change and relative change difference equations are shown below:

*Relative change* (relative Δ pg·mL^−1^) = $\frac{y-x}{x}$ where *x* = the pre-meal resting sample, *y* = the post-meal at the respective sample time-points (i.e., 60, 120 min);

Relative change difference (relative Δ difference pg·mL^−1^) = $\left(\frac{y_{post}-x_{post}}{x_{post}}\right)-\left(\frac{y_{pre}-x_{pre}}{x_{pre}}\right)$ where pre is the pre-intervention results, and post is the post-intervention results.

The data analysis undertaken previously already conforms to these corrected equations. We apologize for this change, which has no impact on the scientific outcomes or conclusions of the study. The original manuscript will remain online on the article webpage, with a reference to this correction.
